# Correction: Synthesis, characterization and biological application of 5-quinoline 1,3,5-trisubstituted pyrazole based platinum(ii) complexes

**DOI:** 10.1039/c8md90005a

**Published:** 2018-02-02

**Authors:** Miral V. Lunagariya, Khyati P. Thakor, Reena R. Varma, Bhargav N. Waghela, Chandramani Pathak, Mohan N. Patel

**Affiliations:** a Department of Chemistry , Sardar Patel University , Vallabh Vidyanagar-388 120 , Gujarat , India . Email: jeenen@gmail.com ; Tel: +912692 226856 218; b Department of Cell Biology , School of Biological Sciences and Biotechnology , Indian Institute of Advanced Research , Koba Institutional Area , Gandhinagar-382007 , Gujarat , India . Tel: +91 79 30514245

## Abstract

Correction for ‘Synthesis, characterization and biological application of 5-quinoline 1,3,5-trisubstituted pyrazole based platinum(ii) complexes’ by Miral V. Lunagariya *et al.*, *MedChemComm*, 2018, DOI: ; 10.1039/c7md00472a.



## 

The authors regret that ligands L^1^–L^5^ are not named correctly in the manuscript. The names are missing ‘4,5-dihydro’ as displayed in the experimental section. The correct names are as follows: 2-chloro-3-(3-(5-chlorothiophen-2-yl)-1-phenyl-4,5-dihydro-1*H*-pyrazol-5-yl)quinoline (L^1^), 2-chloro-3-(3-(4-methylthiophen-2-yl)-1-phenyl-4,5-dihydro-1*H*-pyrazol-5-yl)quinoline (L^2^), 3-(3-(5-bromothiophen-2-yl)-1-phenyl-4,5-dihydro-1*H*-pyrazol-5-yl)-2-chloroquinoline (L^3^), 3-(3-(3-bromothiophen-2-yl)-1-phenyl-4,5-dihydro-1*H*-pyrazol-5-yl)-2-chloroquinoline (L^4^) and 2-chloro-3-(1-phenyl-3-(thiophen-2-yl)-4,5-dihydro-1*H*-pyrazol-5-yl)quinoline (L^5^).

Scheme 1, with corrected name for L^1^–L^5^, is shown below.

**Scheme 1:**
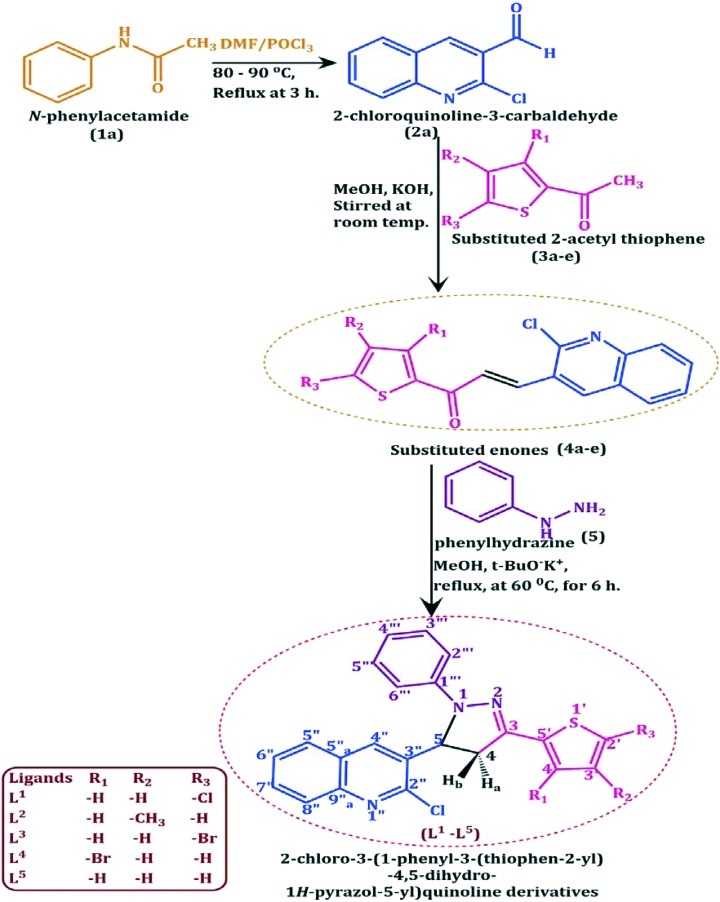


The Royal Society of Chemistry apologises for these errors and any consequent inconvenience to authors and readers.

